# Do Malignant Bone Tumors of the Foot Have a Different Biological Behavior than Sarcomas at Other Skeletal Sites?

**DOI:** 10.1155/2013/767960

**Published:** 2013-03-20

**Authors:** M. Brotzmann, F. Hefti, D. Baumhoer, A. H. Krieg

**Affiliations:** ^1^Orthopaedic Department, Basel University Childrens Hospital (UKBB), Spitalstrasse 33, 4056 Basel, Switzerland; ^2^Bone Tumor Reference Center at the Institute of Pathology, University Hospital Basel, 4031 Basel, Switzerland

## Abstract

We analyze the delay in diagnosis and tumor size of malignant bone tumors of the foot in a retrospective study. We compared the oncological and surgical long-term results with identical tumor at other anatomical sites in order to analyze the biological behavior of sarcomas that are found in the foot. Thirty-two patients with a histologically proven malignant bone tumor (fifteen chondrosarcomas, nine osteosarcomas, and eight Ewing sarcomas) between the years 1969 and 2008 were included. The median follow-up was 11.9 years. The overall median time gap between the beginning of symptoms and diagnosis in the study group was 10 months. Ewing sarcoma presented with the longest delay in diagnosis (median of 18 months), followed by osteosarcoma (median of 15 months) and chondrosarcoma (median of 7.5 months). The delay in diagnosis of these tumors was significantly longer than that of equivalent tumors at other skeletal sites, but the 5- and 10-year survival rates and the occurrence of distant metastases were comparable. In contrast, the average size of foot tumors was 5- to 30-fold less than that of tumors analyzed at other skeletal sites. This study indicates that sarcomas of the foot demonstrate a distinct biological behavior compared to the same tumor types at other skeletal sites.

## 1. Introduction

Bone tumors of the foot are rare and represent only 3%–6% of all bone tumors [1–5]. They are benign in 75%–85% of cases and malignant in 15%–25% [2, 5, 6]. The bone most commonly affected is the calcaneus, followed by metatarsal and phalangeal bones [1, 7]. Chondrosarcoma is the most frequent malignant tumor of the foot, followed by Ewing sarcoma and osteosarcoma [1, 2]. Although there is no thick soft tissue layer to potentially cover a developing mass, a relatively long delay in diagnosis has been reported for such tumors [8]. However, despite a high rate of misdiagnoses, which may lead to incorrect first-line treatment, foot sarcomas rarely develop metastases [5, 9]. It was hypothesized that this might be due to a less aggressive behavior of bone tumors at the foot compared to other sites of the skeletal system [7, 9].

Although amputation of the foot is hardly an acceptable surgical solution for many patients with sarcomas, the resection margins commonly contain residual tumor tissue after initial excision and biological reconstruction. The desire to make a functionally optimal reconstruction and the complexity of this anatomical region can easily lead to an inadequate resection. Wide surgical margins, however, are an important factor for the oncological outcome of malignant bone tumors [9, 10].

The aim of this retrospective study was to evaluate the delay in diagnosis, the tumor size, and the long-term survival rate of patients with malignant bone tumors of the foot. To our knowledge, there is a lack of information regarding these factors in the literature. The results were compared with data from equivalent tumors in the literature both at the foot and also at other skeletal sites.

## 2. Materials and Methods

After approval of the local ethical committee (Reference no. EK 143/08), we retrieved records of 32 patients diagnosed between 1969 and 2008 with a primary bone tumor of the foot from the database of the Bone Tumor Reference Center (BTRC) in Basel. The dataset included age, gender, histology, grade, anatomical site, size (volume) of the tumor, metastases, recurrence, and treatment modalities. In order to obtain detailed information on the chronology of symptoms and patient survival rate, a questionnaire was sent to the patients' general physicians. All patient data are provided in Table 1.

We distinguished between low- (G1) and high-grade (G2 + G3) sarcomas, and all diagnoses were confirmed by a reference pathologist. The tumor volume was calculated roughly respecting its geometrical shape (ellipsoidal or cylindrical) from plain X-rays and computed tomography (CT)/magnetic resonance imaging (MRI) scans, depending on the tumor configuration and presence/absence of a soft tissue component. The interval between diagnosis and the events local recurrence-free survival (LRFS) and metastasis-free survival (MFS) were calculated. Delay in diagnosis was defined as the time period between the first clinical symptoms and the diagnosis, which was based on histology after biopsy.

Adequate treatment of high-grade tumors was considered to be bioptic diagnosis followed by neoadjuvant chemotherapy (in cases of Ewing sarcoma and osteosarcoma) and wide or radical resection (for all kinds of sarcomas). Intralesional resections were considered to be inadequate treatment in all cases. Surgical procedures were classified as radical, wide, marginal, and intralesional, according to Enneking's classification [10].

Data analysis was performed using SPSS 11.5 software (SPSS Inc., Chicago, IL, USA). Data description was primarily based on median and quartile values for continuous endpoints. Binary endpoints were characterized by frequencies. Interindividual comparisons between patient subgroups were based on the two-sample Wilcoxon test for continuous endpoints and Fisher's exact test for binary endpoints. Survival analysis was based on the Kaplan-Meier method and Logrank test. In addition to the overall survival rate (OS), LRFS and MFS were calculated as a function of various clinical parameters. *P* values < 0.05 were considered statistically significant.

## 3. Results

### 3.1. Delay in Diagnosis

The overall median delay in diagnosis of our cases was 10 months (IQR 3–18 months, range 3–128 months). Ewing sarcoma showed the longest delay between onset of symptoms and diagnosis (Table 2). Patients with a delay in diagnosis of >12 months and <12 months did not show a significant difference in the 5-year (86% versus 74%) and 10-year (63% versus 54%) survival rates (*P* = 0.24).

The rate of metastasis when correlated to a delay in diagnosis of >6 or <6 months and >12 or <12 months revealed no significant influence of the delay in diagnosis on the occurrence of subsequent metastasis (*P* = 0.69 for 6 months and 0.44 for 12 months).

### 3.2. Tumor Size, Survival Rate, and Treatment

#### 3.2.1. Chondrosarcoma

The median size of the low-grade chondrosarcomas of the foot was 3.1 mL (IQR 2.0–4.5 mL, range 1.2–158 mL), and all patients with low-grade chondrosarcomas (*n* = 9) were alive at last follow-up. The 5- and 10-year survival rates of these patients were 100% and 86%, respectively (Table 3).

High-grade chondrosarcomas (*n* = 6) had a median size of 16.7 mL (IQR 4–18, range 0.9–45) and showed a 66% (*n* = 4) patient overall survival rate. The 5- and 10-year survival rates of these patients were 83% and 66%, respectively. Patients with chondrosarcomas undergoing radical surgery had significantly better 5- and 10-year survival rates than patients undergoing other surgical treatments (*P* < 0.01).

Two patients with high-grade chondrosarcoma treated with intralesional resection had local recurrences and subsequently amputation in both cases. Both patients died of metastatic disease.

#### 3.2.2. Ewing Sarcoma

The overall survival in patients with Ewing sarcoma was 37.5%, including two patients with no evidence of disease (NED) and one patient alive with disease (AWD). The median size was 14.4 mL (IQR 4.5–36, range 0.9–60). The 5- and 10-year survival rates were 71% and 28%, respectively (Table 3). All patients (*n* = 8) were treated with neoadjuvant chemotherapy according to the current protocols.

Two patients with Ewing sarcoma presented with metastases at the time of diagnosis. In one patient, chemotherapy and surgical treatment of the metastases were successful. The second patient developed recurrent metastases after 55 months, received radiotherapy, and died 2 months later.

The remaining six patients developed distant metastases after a median of 42 months (range 8–70). One patient died 2 months after occurrence of systemic spread without further treatment. Three patients were treated with chemotherapy and the remaining two with radiotherapy following surgery. Five of these six patients died after a median of 8 months (range 2–30). The one surviving patient was treated by resection of the lung metastases and additional chemotherapy.

There were two local recurrences, one of which appeared after a marginal and the second after a radical resection. These patients were treated with amputation or radiotherapy, and both died of metastatic disease.

#### 3.2.3. Osteosarcoma

The overall survival rate of patients with low-grade osteosarcoma (*n* = 4) was 75%, and the median tumor size was 50 mL (IQR 8–101, range 2.5–134). Both 5- and 10-year survival rates of these patients were 67% (Table 3). The only nonsurvivor of this group developed metastatic disease after 7 months and died 19 months later.

The median size of high-grade osteosarcomas of the foot (*n* = 5) was 14.4 mL (IQR 4.5–36, range 3–280). The overall survival rate was 40%, with 5- and 10-year survival rates of 80% and 60%, respectively.

None of the patients with osteosarcoma presented with metastases at the time of diagnosis. After a median of 39 months (IQR 15.3–60, range 4–63), a total of five patients developed distant metastases. In three cases, local surgery was performed, and in the remaining cases, chemotherapy was applied. Only one patient treated surgically was still alive after follow-up of 11.5 years, and the other patients died from metastatic disease. All osteosarcoma patients without metastases were still alive at the time of the latest follow-up.

One patient with low-grade osteosarcoma developed local recurrence after an intralesional resection and was further treated with amputation. The patient refused to undergo the recommended chemotherapy. The patient is still alive without significant impairment of his daily activities.

### 3.3. Local Recurrence

Patients treated with radical resection (*n* = 22) had better 5- and 10-year survival rates compared to those treated with local resection (*n* = 10): 87% versus 72% and 63% versus 49% (*P* = 0.62).

Local recurrence was found in five patients (15.6%) after a median of 8 months (IQR 5.5–18, range 4–48). We found one local recurrence in the group treated with radical resection (Ewing sarcoma). Local recurrence was associated with an adverse outcome and showed a statistically significant influence on 5- (40% versus 90%) and 10-year (20% versus 68%) survival rates (*P* = 0.043).

Four of five patients with local recurrence received inadequate prior treatment. In only one case, a local recurrence occurred despite adequate therapy. Three patients developed subsequent distant metastases.

### 3.4. Overall Treatment

Twenty-three patients (72%) underwent adequate treatment. Of the nine patients receiving inadequate therapy, 7 received insufficient local resection (intralesional/marginal resection). The latter comprised 2 low-grade chondrosarcomas and 5 high-grade sarcomas (2 chondrosarcoma, 1 osteosarcoma, and 2 Ewing sarcomas). One patient with osteosarcoma refused to undergo chemotherapy, and one patient with Ewing sarcoma had an inadequate neoadjuvant chemotherapy.

### 3.5. Metastases

Twelve patients developed distant metastases after a median of 27.5 months (IQR 13–51.3, range 3–70). Four patients presented with metastases already at the time of diagnosis (*P* = 0.039). Patients with metastases at the time of diagnosis had worse 5- and 10-year survival rates (40% and 20%) than those without (89% and 65%). Patients with late metastases had a significantly lower survival rate compared to patients without metastases (58% versus 100% after 5 years and 17% versus 88% after 10 years; *P* = 0.01).

## 4. Discussion

In the recent years, we have observed several patients with malignant bone tumors of the foot with a long delay in diagnosis. In this study, we wanted to elucidate whether such a delay may reflect characteristic biological differences between bone sarcomas of the foot and their counterparts at other skeletal sites. To our knowledge, there is only one study in the literature reporting on the delay in treatment of tumors of the foot but not in comparison to tumors at other sites of the skeletal system [1].

Because the foot has only a thin soft tissue envelope, one would suspect swelling caused by a tumor to lead to an immediate clinical recognition. However, we observed a long overall delay in diagnosis in the foot, especially in high-grade tumors.

Ewing sarcomas, which usually are rapidly and aggressively growing lesions, showed the longest delays (median of 18 months). This is 2–6 times longer than delays in Ewing sarcomas located at other sites of the skeleton [2, 11–13]. These findings are consistent with Adkins et al. [14] and Metcalfe and Grimer [15] reporting on a delay of 11,75 and 14 months. In addition, the sizes of sarcomas in our patients were considerably smaller than those at other sites.

Delays seen in diagnosis of osteosarcomas in our study (median of 15 months), as with Ewing sarcomas, were considerably longer (4.5- to 14-fold) than reported for osteosarcomas at other sites [2, 11, 12, 16, 17]. Likewise, the volume of these tumors was much smaller than reported for tumors at other sites.

In contrast to Ewing sarcomas, the more slowly growing chondrosarcomas showed the shortest median delay in diagnosis with 7.5 months. This is almost comparable to the delay in diagnosis of chondrosarcomas at sites other than the foot.

Several authors argue that the rarity of bone tumors in this special anatomical location is a major cause for the long delay in diagnosis of bone tumors of the foot [8, 9, 18]. In our opinion, this argument is not very convincing, since bone tumors are rare anyway. First symptoms as pain and swelling are unspecific and frequently misinterpreted as being of inflammatory or posttraumatic nature. The variety of differential diagnoses explains the long delay in diagnosis of bone tumors in general but not the striking difference between tumors of the foot and those at other skeletal sites.

Zeytoonjian et al. [9] tumors found a death rate of 8% in primary malignant bone tumors of the foot compared to 27% in tumors in other anatomical locations.

In this study, the death rate (34%) was higher but in the same range of sarcomas at other sites. However, despite the higher death rate, the long delay, and a relatively large proportion of cases with inadequate treatment, the OS is not significantly worse. It has been assumed that primary malignant bone tumors of the foot may have less deleterious effects than those located at other sites, but this is not completely understood [1, 3, 4, 9, 19]. Results of our study indicate that these tumors in foot may have certain basic biological differences from those at other sites.

The delay in diagnosis of primary malignant bone tumors of the foot is—especially for high-grade tumors—considerably longer than that at other sites (Table 2). In contrast, the average volume is tumors significantly smaller than reported for other sites (Table 4). For chondrosarcomas localized in the rest of the skeleton, the size is 20–30-fold, for osteosarcomas 3–10-fold, and for Ewing sarcomas 5-6-fold higher according to the literature [20–24]. The difference is even more striking if the time of development is taken into consideration. Based on these assumptions, a rough calculation of 12-month tumor development in chondrosarcomas would, for example, result in a tumor volume of 30 mL at the foot and of 800 mL at other sites. Although the evidence of such estimations is not very strong, the difference is so obvious that it allows the assumption that tumors of the foot exhibit a different biological behavior and grow much slower. This could explain the long delays in diagnosis. The survival rate of malignant bone tumors of the foot is affected by metastases at the time of diagnosis, the occurrence of distant metastasis, and local recurrence of the primary tumor. In these respects, sarcomas in the foot do not differ from those at other sites. Such factors normally significantly worsen the prognosis, but this was not found in our study. However, the long delay in diagnosis found in this study did not correlate with a higher rate of primary metastases.

The risk of developing a local recurrence is eight times higher with an inadequate compared to an adequate therapy. Local recurrence is associated with a significantly decreased survival rate and a higher occurrence of metastases. Consequently, the prognosis worsens. In our series, there was a significantly lower survival rate for patients with distant metastases (*P* = 0.01). In summary, patients with a local recurrence have a worse survival rate, accompanied by a higher rate of distant metastases. This phenomenon is well known in the literature too [19, 25].

As expected, comparing adequate versus inadequate treatments indicated a positive influence of adequate treatment on the survival rates in this study. These rates imply an unequivocal but not significantly better prognosis (*P* = 0.26). One reason for the high number of patients with an inadequate treatment is the long follow-up of the study; diagnoses and treatments were performed in the 1970s and 1980s. Meanwhile, the treatment regimens have markedly changed—for example, multimodal therapy regimes including chemotherapy—and have led to significant improvements in the outcome for patients with sarcomas [2, 3, 14, 19, 26–28].

The main cause for an inadequate therapy was an insufficient surgical procedure. In 7 of 9 patients with inadequate therapy in our study, an intralesional or marginal resection was performed most likely caused by the specific anatomical challenges in this location (e. g. small compartments). Compared to intralesional, marginal, or wide resections, we found a significantly lower rate of local recurrences and higher survival rates in patients who underwent radical surgical treatment. This is in accordance with the results of other studies considering radical surgery as the best option for local tumor control too [9, 18, 28, 29]. Despite radical resection, patients with foot sarcomas usually do not have significant functional restrictions after surgery and rehabilitation.

An unknown factor is the latency of the tumor (i.e. time between the emergence of the first tumor cell and the appearance of symptoms). It is quite probable that the latency of sarcomas of the foot is shorter than that at other sites. In such a case, tumors in long bones and the trunk would be larger at clinical manifestation than comparable tumors of the foot. Likewise, the longer latency at other sites could be attributed to a masking by the relatively thick soft tissue layers in the leg and trunk. The indeterminacy of latency is a weakness in the calculation of tumor growth before diagnosis. As cell growth is exponential, detectable increases in tumor volume require much more time in small compared to large tumors. Nevertheless, the observed differences between tumor growth in the foot and other sites are striking. It is likely that—despite the unknown latency factor—this reflects a differential biological behavior.

One major limitation of this study is that almost one half of the patients were diagnosed and treated before the end of the 1980s when chemotherapeutic regimes and imaging modalities were improved dramatically. Further limitations derive from the retrospective design and the small patient population. Sarcomas of the foot are rare, but the number of patients in this series is within the range (6–87 patients) of those in other reports [1, 3, 6, 8, 15]. In contrast, the long median follow-up of 11.9 years is the strength of this study.

In conclusion, primary malignant bone tumors of the foot appear to grow slower and to be less aggressive than those at other anatomical locations. We observed a long delay in diagnosis of foot sarcomas, which is in contrast to the general assumption that the thin soft tissue layer of the foot should allow an immediate clinical recognition. Interestingly, despite the delay in diagnosis, the prognosis is similar to that of tumors at other skeletal locations. From a systematic comparison of reported delays in diagnosis and tumor volumes at other sites, we conclude that malignant tumors of the foot grow at an approximately 10–20-fold slower rate than tumors at other sites of the body, and this property indicates a distinct biological behavior of bone tumors in this special anatomic location.

## Figures and Tables

**Table 1:** Patient data.

ID number/ gender	Tumor	Grading G1–G3^1^	Age^2^	Tumor size^3^ (volume^4^)	Tumor site	Primary metastasis	Late metastasis time^5^	Delay in diagnosis^5^	Operative treatment^6^	Recurrence/ time^5^	Survival time^2^	Follow-up^2^	Adequate therapy	Status^7^
1/♀	Chondrosarcoma	G 1	59.5	1.5 × 2.0 × 1.0 (3.0)	Calcaneus	No	No	6.0	4	No	13.91	13.91	No	NED
2/♀	Chondrosarcoma	G 2	72.6	4.0 × 1.0 × 1.0 (4.0)	Phalanx Dig. IV	No	No	Unknown	1	No	15.58	15.58	Yes	DOC
3/♂	Chondrosarcoma	G 1	72.9	3.4 × 3.0 × 4.0 (40.8)	Calcaneus	No	No	1.0	4	No	9	9	No	DOC
4/♀	Chondrosarcoma	G 1	52.3	1.5 × 1.0 × 1.0 (1.5)	Phalanx Dig. III	No	No	Unknown	1	No	8.58	8.58	Yes	NED
5/♂	Chondrosarcoma	G 2	22.0	1.2 × 0.8 × 1.0 (0.9)	Phalanx Dig. II	No	No	3.0	1	No	12.25	12.25	Yes	NED
6/♂	Chondrosarcoma	G 1	66.7	1.8 × 1.4 × 1.2 (3.0)	Dig. V	No	No	128.0	1	No	18.25	18.25	Yes	NED
7/♀	Chondrosarcoma	G 1	36.4	2.0 × 1.6 × 1.0(3.2)	Os metatarsale II	No	No	10.0	1	No	8.83	8.83	Yes	NED
8/♂	Chondrosarcoma	G 1	66.7	6.5 × 6.1 × 4.0 (158.0)	Calcaneus	No	No	9.0	3	No	11.91	11.91	Yes	NED
9/♀	Chondrosarcoma	G 1	39.5	2.0 × 1.5 × 1.5 (4.5)	Phalanx Dig. I	No	No	5.0	1	No	26.33	26.33	Yes	NED
10/♂	Chondrosarcoma	G 2	62.5	Unknown	Calcaneus	Yes	No	10.00	4	Yes/8	6.91	—	No	DOD
11/♀	Chondrosarcoma	G 2	67.6	3.0 × 6.0 × 1.0 (18.0)	Os metatarsale II	No	No	13.0	1	No	8.66	8.66	Yes	NED
12/♂	Chondrosarcoma	G 1	28.1	2.0 × 1.0 × 1.0 (2.0)	Phalanx Dig. III	No	No	1.0	1	No	13.83	13.83	Yes	NED
13/♂	Chondrosarcoma	G 2	20.0	2.5 × 2.5 × 2.7 (16.7)	Phalanx Dig. III	No	No	Unknown	1	No	7.41	7.41	Yes	NED
14/♂	Chondrosarcoma	G 3	68.9	3.0 × 5.0 × 3.0 (45.0)	Calcaneus	Yes	Yes/3	9.0	4	Yes/4	0.5	—	No	DOD
15/♂	Chondrosarcoma	G 1	29.4	1.2 × 1.0 × 1.0 (1.2)	Phalanx Dig. III	No	No	1.0	1	No	20.83	20.83	Yes	NED
16/♀	Osteosarcoma (central chondroblastic)	G 2	39.4	4.0 × 1.8 × 2.0 (14.4)	Os metatarsale IV	No	Yes/63	1.0	1	No	11.5	11.5	Yes	NED
17/♀	Osteosarcoma (parosteal)	G 2	69.3	3.0 × 1.0 × 1.0 (3.0)	Os metatarsale I	No	Yes/4	4.0	1	No	2.6	—	Yes	DOD
18/♂	Osteosarcoma (parosteal)	G 1	43.3	4.0 × 2.5 × 1.0 (10.0)	Os metatarsale I	No	No	9.0	2	No	2.0	2.0	Yes	NED
19/♀	Osteosarcoma (central)	G 1	22.8	8.0 × 4.2 × 4.0 (134.0)	Calcaneus	No	No	16.0	4	Yes/6	17.58	17.58	No	NED
20/♂	Osteosarcoma	G3	49.8	3.0 × 3.0 × 4.0 (36.0)	Phalanx Dig. I	No	Yes/59	18.0	1	No	25.00	—	No	DOD
21/♀	Osteosarcoma (central)	G 1	57.5	6.0 × 6.0 × 2.5 (90.0)	Talus	No	Yes/7	19.0	1	No	2.33	—	Yes	DOD
22/♀	Osteosarcoma	G 3	14.8	7.0 × 8.0 × 5.0 (280.0)	Os metatarsale I	No	No	15.0	1	No	10.08	10.08	Yes	NED
23/♀	Osteosarcoma	G 2-3	45.1	3.0 × 1.5 × 1.0 (4.5)	Phalanx Dig. I	No	Yes/19	2.0	1	No	6.08	—	Yes	DOD
24/♀	Osteosarcoma (central)	G 1	47.0	2.5 × 1.0 × 1.0 (2.5)	Os metatarsale II	No	No	23.0	1	No	8.5	8.5	Yes	NED
25/♂	Ewing sarcoma	G 3	19.0	3.5 × 3.5 × 1.0 (12.3)	Calcaneus	No	Yes/8	Unknown	1	Yes/ unknown	2.58	—	Yes	DOD
26/♂	Ewing sarcoma	G 3	11.4	4.0 × 3.5 × 3.2 (45.0)	Calcaneus rechts	No	No	5.0	4	No	18.25	18.25	No	NED
27/♀	Ewing sarcoma	G 3	16.8	4.0 × 1.2 × 1.0 (4.8)	Os metatarsale III	No	Yes/70	11.0	1	No	6.41	—	No	DOD
28/♂	Ewing sarcoma	G 3	9.8	1.2 × 0.8 × 1.0 (0.9)	Os metatarsale I	Yes	No	34.0	2	No	11.91	11.91	Yes	NED
29/♂	Ewing sarcoma	G 3	18.8	4.0 × 1.5 × 1.5 (9.0)	Os metatarsale IV	Yes	Yes/55	24.0	3	Yes/48	4.25	—	No	DOD
30/♂	Ewing sarcoma	G 3	11.6	5.0 × 3.0 × 4.0 (60.0)	Os metatarsale I	No	Yes/49	3	1	No	5.91	—	Yes	DOD
31/♂	Ewing sarcoma	G 3	51.7	3.0 × 4.0 × 4.0 (48.0)	Os metatarsale IV	No	Yes/36	26.0	1	No	5.5	—	Yes	DOD
32/♀	Ewing sarcoma	G 3	17.7	4.3 × 2.9 × 1.9 (24.0)	Calcaneus	No	Yes/15	18.0	1	No	3.0	3.0	Yes	AWD

^1^Low grade = G1; high grade = G2/G3; ^2^age in years; ^3^size in cm; ^4^volume in mL; ^5^in months; ^6^radical = 1; wide = 2; marginal = 3; intralesional = 4; ^7^DOC: death of other cause; DOD: death of disease; AWD: alive with disease; NED: no evidence of disease.

**Table 2:** Delay in diagnosis at the foot (the present study) and at the other sites (the literature).

Time of delay in diagnosis (in months)		
Diagnosis	Median results of the present study	Average results from other sites in the literature
Chondrosarcoma	7.5 (IQR 1.5–12.2, range 1–128)	10 (G1-G2)
		5 (G3) [25]

Osteosarcoma	15 (IQR 3–18.5, range 1–23)	3.5 [17]
		5.2 [30]
		6.4 [11]

Ewing sarcoma	18 (IQR 5–26, range 3–34)	8.5 [12]
		9.6 [11]
		8.1 [30]
		3–9 [13]

Abbreviations used: IQR–interquartile range. The superscripts listed in the last column of the table refer to references.

**Table 3:** Five- and 10-year survival rates of sarcomas of the foot compared with rates at other skeletal sites.

Diagnosis		Results of the present study		Results from other sites in the literature	
	Grading	5-year survival rate	10-year survival rate	5-year survival rate	10-year survival rate
Chondrosarcoma	G1 (*n* = 9)	100%	86%	89%–96% [25, 31]	89% [25]
	G2/G3 (*n* = 6)	83%	66%	53%–62% [25, 31]	38%–53% [25]

Osteosarcoma	G1 (*n* = 4)	67%	67%	66% [32]	—
	G2/G3 (*n* = 5)	80%	60%	60%–80% [26, 31]	20%–49% [26, 31]

Ewing sarcoma	G3 (*n* = 9)	71%	28%	50%–70% [19, 27, 31]	20%–50% [19, 27, 31]

The superscripts listed in the last two columns of the table refer to references.

**Table 4:** Average volume of tumors in the foot and at other anatomical sites.

Median/average volume of tumor at diagnosis		
Diagnosis	Median volume in the present study	Average volume from other sites reported in the literature
Chondrosarcoma	21.2	400 [20]
		600 [25]

Osteosarcoma	63.8	182 [21]
		650 [22]
		242 [23]

Ewing sarcoma	25.5	145 [27]
		144 [24]

The superscripts listed in the last column of the table refer to references.
